# “You can’t put your head down like an ostrich” - Emotional experiences associated to clozapine treatment protocol reported by patients with schizophrenia seen in a Brazilian university specialized service: a clinical-qualitative study

**DOI:** 10.1192/j.eurpsy.2023.594

**Published:** 2023-07-19

**Authors:** E. R. Turato, J.-B. A. Santos, P. Dalgalarrondo, C. R. Dantas, F.-C. Specian-Jr, L. S. Valladão

**Affiliations:** Laboratory of Clinical-Qualitative Research, State University of Campinas, Campinas, Brazil

## Abstract

**Introduction:**

Understanding the psychological meanings of a rigorous protocol for introducing a drug to patients is a challenge of emotional management for clinical professionals. Clozapine is an effective drug for patients with schizophrenia resistant to treatment with first and second-generation antipsychotics. This medication has agranulocytosis as an important side effect. The medication protocol requires frequent blood draws to monitor any effect on blood cells. Investigating patients’ emotional perceptions about the experience with this procedure was the triggering question of our study.

**Objectives:**

To interpret emotional/symbolic meanings attributed by patients with schizophrenia to the protocol of introduction of clozapine in follow-up at a specialized university service.

**Methods:**

Clinical-qualitative design of Turato. Semi-directed interviews with open-ended questions in-depth were conducted face-to-face with participants using clozapine. Closed sample by the theoretical information saturation criterion described by Fontanella. Data were treated by Faria-Schutzer’s Clinical-Qualitative Content Analysis, employing psychodynamic concepts of the theoretical framework of Medical Psychology.

**Results:**

From the analysis of nine patients, three categories emerged: 1) “Anyway, I come here to stay alive”: frequent blood collections of the protocol seem to have a good impact on patient’s adherence to treatment; 2) To re-signify a psychiatric illness: the protocol reinforcing an embodiment of a medical diagnosis; 3) “It is a very big precaution”: the protocol as real and emotional support to deal with the possibility of serious side effects.

**Conclusions:**

Although blood collection is a repetitive experience for patients, such a routine does not mean that the procedure brings symbolizations that are more charged than the disease itself. Patients can benefit from the commitment to attend blood collection frequently, as it removes them from possible social isolation, allowing social interaction; it brings the perception of emotional security due to the commitment to the clinical team. These benefits can lead the patient to develop new meanings for their life condition. Future qualitative research can be conducted to study the meanings of medical protocols in other diagnostic situations.

**Disclosure of Interest:**

None Declared

